# BMI as a Predictor for Potential Difficult Tracheal Intubation in Males

**DOI:** 10.3389/fmed.2015.00038

**Published:** 2015-06-04

**Authors:** Alberto A. Uribe, David A. Zvara, Erika G. Puente, Andrew J. Otey, Jianying Zhang, Sergio D. Bergese

**Affiliations:** ^1^Anesthesiology, The Ohio State University Wexner Medical Center, Columbus, OH, USA; ^2^The University of North Carolina, Chapel Hill, NC, USA; ^3^Center for Biostatistics, The Ohio State University, Columbus, OH, USA; ^4^Neurological Surgery, The Ohio State University Wexner Medical Center, Columbus, OH, USA

**Keywords:** obesity, difficult, tracheal intubation, predictors, BMI

## Abstract

**Introduction:**

Difficult tracheal intubation is a common source of mortality and morbidity in surgical and critical care settings. The incidence reported of difficult tracheal intubation is 0.1%–13% and reaches 14% in the obese population. The objective of our retrospective study was to investigate and compare the utility of body mass index (BMI) as indicator of difficult tracheal intubation in males and females.

**Material and methods:**

We performed a retrospective chart review of patients who underwent abdominal surgeries with American Society of Anesthesiologists I to V under general anesthesia requiring endotracheal intubation. The following information was obtained from medical records for analysis: gender, age, height, weight, BMI, length of patient stay in the Post Anesthesia Care Unit, past medical history of sleep apnea, Mallampati score, and the American Society of Anesthesiologists classification assigned by the anesthesia care provider performing the endotracheal intubation.

**Results:**

Of 4303 adult patients, 1970 (45.8%) men and 2333 (54.2%) women were enrolled in the study. Within this group, a total of 1673 (38.9%) patients were morbidly obese. The average age of the study group was 51.4 ± 15.8 and the average BMI was 29.7 ± 8.2 kg/m^2^. The overall incidence of the encountered difficult intubations was 5.23% or 225 subjects. Thus, our results indicate that BMI is a reliable predictor of difficult tracheal intubation predominantly in the male population; another strong predictor, with a positive linear correlation, being the Mallampati score.

**Conclusion:**

In conclusion, our data shows that BMI is a reliable indicator of potential difficult tracheal intubation only in male surgical patients.

## Introduction

The difficult tracheal intubation is a common source of mortality and morbidity in surgical and critical care settings. The incidence reported in literature varies between 0.1% and 13% (1–3) and reaches 14% in the obese population (4). The adverse events related to difficult tracheal intubation include, but are not limited to: hypoxic brain injury, cardiopulmonary arrest, rescue tracheostomy, airway trauma, aspiration, damage to teeth, and death (1). Various parameters have been studied in an attempt to establish a better predictor of potential difficult intubation. However, there is no strong consensus and the results are still unclear on true predictors and criteria to be used to predict potential difficult laryngoscopies (5–7). Since the available literature does not have a standard definition of difficult airway, the American Society of Anesthesiologists (ASA) defined it as “the clinical situation in which a conventionally trained anesthesiologist experiences difficulty with mask ventilation, difficulty with tracheal intubation, or both” (4, 8).

The Practice Guidelines for Management of the Difficult Airway recommend acquiring an airway history and performing an examination prior to the initiation of anesthesia and airway manipulation (8). Preoperative assessment of the airway is an essential and standard component of anesthesia care. For the reason of the inherent risk associated with difficult intubations, having an effective and reliable predictor of a difficult airway is essential (2). Anesthesia care providers are currently using numerous scoring tools, systems, and methods designed to predict potential difficult tracheal intubation. These include, but are not limited to, Mallampati and Cormack–Lehane scales, measuring the neck circumference, interincisor gap or thyromental distance, and Intubation Difficulty Score (IDS) (9).

For most people, the body mass index (BMI) correlates with the amount of body fat. According to this index, people are further classified as underweight (<18.5 kg/m^2^), normal (18.5–24.9 kg/m^2^), overweight (25–29.9 kg/m^2^), obese (30–34.9 kg/m^2^), and morbidly obese (>35 kg/m^2^) (4). In recent years, the average BMI of Americans has increased in the young adult population, making obesity a real epidemic (7). According to the Centers for Disease Control and Prevention, a study done in 2007–2008 approximates that one-third (33.8%) of American adults is obese compared to 22.9% in 1988–1994. Considering an increased rate of difficult intubations in obese patients, it will be important to answer the clinically relevant question of whether increased BMI will correlate with the level of difficulty of endotracheal intubation and, thus, be a valuable indicator of a possible difficult intubation (4).

The objective of our retrospective study was to investigate and compare the utility of BMI as an indicator of difficult tracheal intubation in males and females and to determine whether it can be reliably used in clinical settings as a predictor of potential difficult intubation.

## Materials and Methods

We conducted a retrospective review of randomly selected medical records of patients who underwent abdominal surgeries and required general anesthesia. After obtaining the approval of the Institutional Review Board, a computerized search was initiated through the electronic medical records, which revealed 20,985 patients who underwent abdominal surgery requiring general anesthesia at The Ohio State University Wexner Medical Center during a period of 12 months, from January 1, 2007 to December 31, 2007. Using Microsoft Excel, every third patient from an alphabetized list was selected to generate a random sample of 6964 patients for this study. We performed a retrospective chart review of patients who underwent abdominal surgeries with ASA stratification I–V under general anesthesia requiring endotracheal intubation. All the patients aged <18 or >85 years, pregnant women, prisoners, patients that required laryngeal mask airway, tracheal stoma, and/or nasotracheal intubation were excluded from the study. The following information was obtained from medical records for analysis: gender, age, height, weight, BMI, length of patient stay in the Post Anesthesia Care Unit (PACU), past medical history of sleep apnea, Mallampati score, and the ASA classification assigned by the anesthesia.

## Statistical Analysis

In a univariate analysis, Pearson chi-square test or Fisher’s exact test were used to evaluate the categorical data – BMI categories, Mallampati score, ASA score, the incidences of sleep apnea, and difficult intubation. Student’s *t*-test or simple logistic regression was used to evaluate the normally distributed numeric variables such as the patient age or length of PACU stay. Using a multivariate analysis, the logistic regression model, including all risk factors, was used to identify the significantly correlated factors when confounding with other factors. The interaction term BMI for male and female patients was also included for stratified analysis of BMI. The Hosmer–Lemesshow test was performed for goodness of fit analysis of the logistic regression model. Odds ratios, and their 95% confidence intervals (CI) were calculated for BMI and Mallampati scores, both before and after the adjustment for other confounders.

## Results

**Figure 1:**
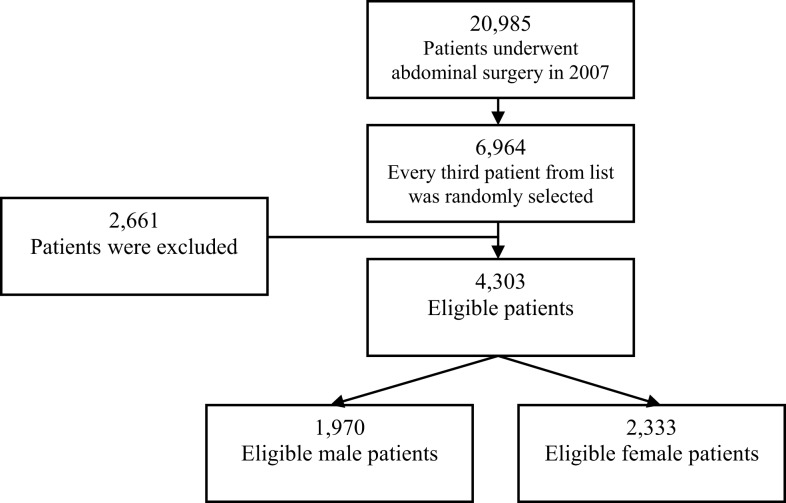
**Patient screening and sample population flow chart**.

Of 6964 patients randomly selected, 2661 (38.2%) patients were excluded from the study due to not meeting the inclusion criteria or missing essential information in their medical records for data analysis. As a result, 4303 adult patients, 1970 (45.8%) men and 2333 (54.2%) women, were enrolled in the study. Figure 1 illustrates the patient screening and sample population flow chart. Within the eligible patients group, a total of 812 (18.9%) were obese and 861 (20.0%) were morbidly obese (Table 1). The average age of the study group was 51.4 ± 15.8, and the average BMI was 29.7 ± 8.2 kg/m^2^ (Table 2). The overall incidence of encountered difficult intubations was 5.2% (225 subjects) (Table 1). In 150 cases of difficult tracheal intubation (66.7%), no additional equipment had been used. In 75 patients (33%), an alternative intubation technique had been utilized (fiberoptic endoscopy or glidescope-assisted intubation).

**Table 1:** **The incidence of difficult intubation in various body mass index categories**.

BMI	Difficult intubation		Total(*N*)
	NO	YES	
	*N* (%)	*N* (%)	
Underweight: <18.5 kg/m^2^	93 (89.4%)	11 (10.6%)	104
Normal: 18.5–24.9 kg/m^2^	1124 (96.9%)	36 (3.1%)	1160
Overweight: 25–29.9 kg/m^2^	1311 (96.0%)	55 (4.0%)	1366
Obese I: 30–34.9 kg/m^2^	758 (93.3%)	54 (6.7%)	812
Obese II+: >35 kg/m^2^	792 (92.0%)	69 (8.0%)	861
Total	4078 (94.8%)	225 (5.2%)	4303

*P-value for chi-square test is <0.0001, implying that BMI and difficult intubation are highly correlated*.

**Table 2:** **Demographics and predictors of difficult intubation**.

	Difficult intubation	Easy intubation	*P-*value
	*n* = 225 (5.2%)	*n* = 4078 (94.8%)	
Age (year)	55 ± 13.1	51.2 ± 16	<0.0001
**Gender**			
Male	127 (6.4%)	1843 (93.6%)	0.0012
Female	98 (4.2%)	2235 (95.8%)	
Height (cm)	172 ± 10.9	169.9 ± 10.7	0.013
Weight (kg)	95.7 ± 30.2	85.5 ± 24	<0.0001
BMI (kg/m^2^)	32.3 ± 9.8	29.6 ± 8.1	<0.0001
**Mallampati score**			
I	18 (1.5%)	1204 (98.5%)	<0.0001
II	120 (5%)	2278 (95%)	
III	71 (14.6%)	415 (85.4%)	
IV	16 (44.4%)	20 (55.6%)	
**ASA classification**			
I	3 (1.3%)	228 (98.7%)	<0.0001
II	55 (3.6%)	1456 (96.4%)	
III	119 (6.4%)	1738 (93.6%)	
IV	48 (7%)	635 (93%)	
LOS PACU	1.92 ± 1.3	1.87 ± 1.2	0.593
**History of sleep apnea**			
Yes	22 (11.4%)	171 (88.6%)	<0.0001
No	203 (4.9%)	3907 (95.1%)	

*Data are presented as mean ± standard deviation*.

*LOS PACU, length of stay in PACU*.

**Figure 2:**
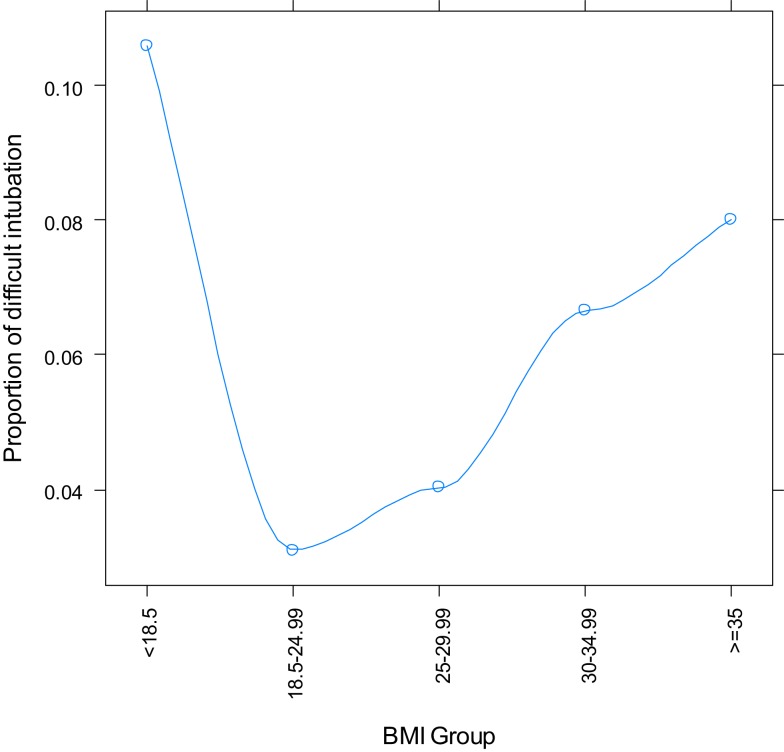
**Proportion of difficult intubation vs. body mass index category**.

**Figure 3:**
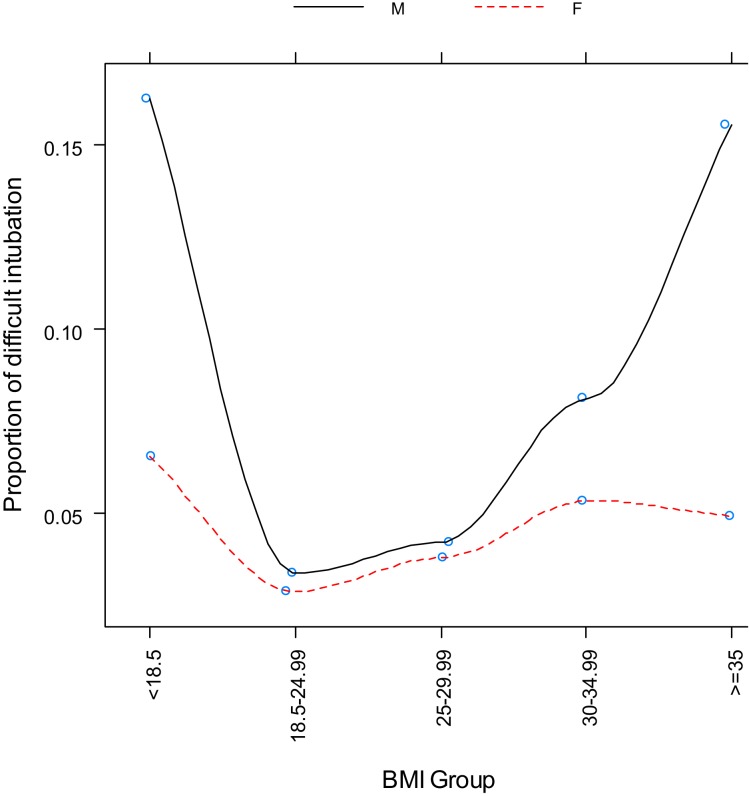
**Proportion of difficult intubation by gender vs. body mass index category**.

**Figure 4:**
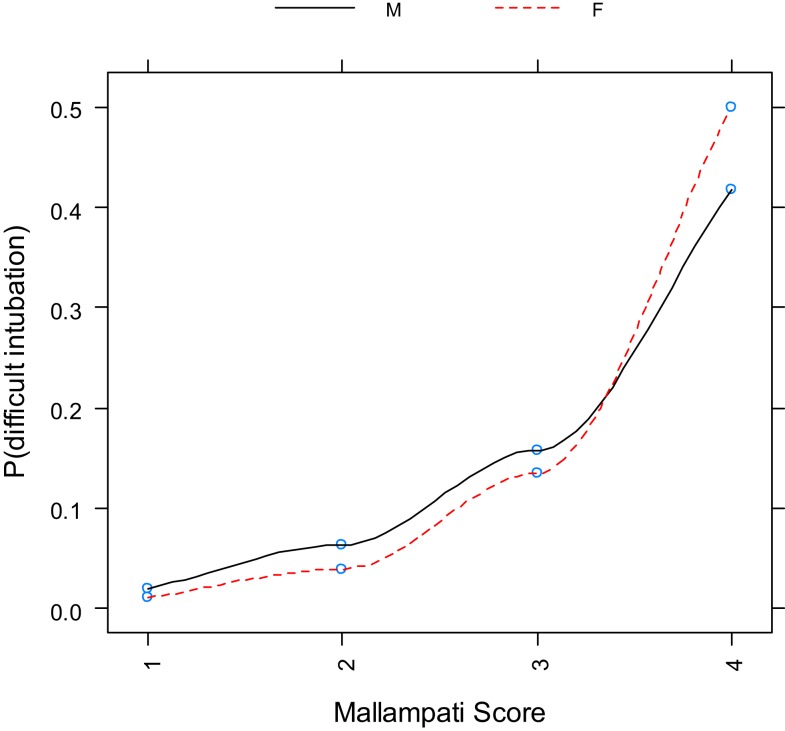
**Proportion of difficult intubation by gender vs. Mallampati score**.

Logistic regression with BMI as a single categorical variable showed that BMI strongly correlated with difficult tracheal intubation (*P* < 0.0001) (Table 3, Figure 2). The odds ratios with 95% CI are presented in Table 4. This overall correlation was further stratified for men and women to determine if the correlation between BMI and difficult tracheal intubation was gender-dependent. In male patients, the proportion with difficult tracheal intubation increased parallel to an increase of the BMI from normal to obese (BMI >35 kg/m^2^) (Table 3). On the other hand, the relationship was weaker in the female group (Figure 3). Therefore, the findings suggest that BMI and gender interacted (*P* = 0.0268) according to the logistic regression. This also suggests that the gender difference was significant among obese (BMI II) patients (Table 3). Thus, our results indicate that BMI could be another predictor of difficult tracheal intubation, predominantly in the male population. Mallampati score was another strong predictor of difficult tracheal intubation with a positive linear correlation (*P* < 0.0001; odds ratio =3.50; 95% CI 2.87–4.28 for every score increase by 1). The correlations for male and female groups were almost equal (Figure 4) with no significant gender-related difference (*P* = 0.1697). We used a full logistic regression model with all the aforementioned risk factors and other confounders fitted. Only the BMI, Mallampati score, and age significantly correlated with difficult tracheal intubation (*P* < 0.0001, *P* < 0.0001, and *P* < 0.002, respectively). There was no strong correlation between the difficult tracheal intubation and the length of patient stay in the PACU, prior diagnosis of sleep apnea, or the ASA group in the full model.

**Table 3:** **Body mass index and difficult intubation stratified by gender**.

BMI	Difficult intubation						*P*-value (male vs. female)
	Male			Female		
	Yes	No	Yes/total (%)	Yes	No	Yes/total (%)	
Underweight: <18.5	7	36	16.28%	4	57	6.56%	0.1124
Normal: 18.5–24.9	18	515	3.38%	18	609	2.87%	0.6202
Overweight: 25–29.9	32	729	4.20%	23	582	3.80%	0.7064
Obese I: 30–34.9	31	351	8.12%	23	407	5.35%	0.1143
Obese II+: >35	39	212	15.54%	30	580	4.92%	<0.0001
Total	45	707	5.98%	37	879	4.20%	

**Table 4:** **Odds ratio estimates and 95% confidence interval for body mass index**.

Effect	Odds ratio	95% CI	
		Lower	Upper
BMI underweight vs. normal	3.695	1.821	7.496
BMI overweight vs. normal	1.31	0.854	2.009
BMI I^a^ vs. normal	2.224	1.444	3.425
BMI II^a^ vs. normal	2.72	1.8	4.111

*^a^BMI I: 30–34.9, BMI II: >35*.

The endotracheal intubations had predominantly been performed by experienced professionals.Most of the difficult tracheal intubation cases (70%) were performed by well-trained anesthesia care providers (44% certified registered nurse anesthetists and 26% physician anesthesiologists), 14% were performed by categorical/advance first and third-year residents of anesthesiology and 16% were unknown because records were not available.

## Discussion

The Mallampati score was developed in 1985 by Mallampati et al., and it is the most commonly used method to predict a difficult tracheal intubation by anesthesia care providers (7). In order to assess the airway, the patient is instructed to open his/her mouth and protrude the tongue for the optimal view of the oral cavity, specifically the base of the uvula, faucial pillars, and soft palate. The score can be done with or without phonation and is graded on a 1–4 scale, where scores of 3 or 4 are generally considered indicators of a difficult airway (7). Due to its subjectivity, the test has proved to be an imperfect predictor of a difficult airway with low inter-rater reliability (10). Another limitation is the lack of consensus on what constitutes a difficult airway.

Many authors rely on the Cormack–Lehane scale first introduced in 1984. The method allows grading the extent of glottic and laryngeal visualization using direct laryngoscopy. Grades of 3 or 4, on a scale of 1–4 are generally related to difficult tracheal intubation (7, 11–15). As an alternative to assess difficult airway, neck circumference, thyromental distance, and interincisor gap measurement have been studied to predict difficult intubation (9). Although, they were found to be significant predictors of difficult intubation, they are not ideal tools because they are not currently part of standard preoperative assessments.

Other studies rely on the IDS developed and introduced by Adnet et al. in 1997 (1). The IDS is a quantitative method to score intubation difficulty post factum; thus, it is more an indicator than a predictor of a difficult intubation. This scale includes seven parameters scored after intubation: the number of alternative techniques used, a modified Cormack grade for glottic visualization (0, complete visualization; 3, no visualization); subjective impression of the lifting force required during laryngoscopy (normal or increased), the need for external laryngeal pressure to optimize glottic exposure (applied or not applied, not counting Sellick’s maneuver done to prevent aspiration), and the position of the vocal cords (abduction or adduction). The score is calculated by summing the 7 criteria labeled N1–N7, where a score greater than 5 is considered an indication of difficult tracheal intubation (9, 16–18). Unlike neck circumference, interincisor, and thyromental distance, and several other measurements, BMI is a parameter recorded in every chart. Unlike the Mallampati score, BMI is a consistent data collected as standard of care in patient’s mass (*M*) and height (*H*) based on the formula: BMI =*M* kg/*H* cm^2^.

In our study, the incidence of difficult intubations among patients with general surgical pathology was 5.23% (2.95% for males and 2.28% for female patients). The incidence of difficult tracheal intubation increased parallel to an increase BMI, Mallampati score, and ASA classification. BMI and Mallampati score were related with difficult intubation as shown in Figure 2. There was a positive correlation between the BMI and percentage of patients with difficult intubations for normal and high BMI, while it became negative for underweight patients (Figure 2). The underweight patients constituted a much smaller group compared to the other groups, making it impossible to obtain conclusive results. A similar incidence of difficult intubations was reported by Lundstrøm et al. (18). Our data confirms the reports in the literature; the BMI may be a reliable predictor of difficult intubation (7). Based on our results, it is a strong predictor for difficult laryngoscopy in men, but not women. In contrast, Mallampati score grades II–IV are reliable indicators of possible difficult tracheal intubation for both genders.

There are conflicting reports regarding the correlation of BMI and difficult intubation (4, 18). Ezri et al. (2002) found that head and neck movements, high arched palate, thyromental distance, and a modified Mallampati test are better predictors for difficult tracheal intubation than overall BMI (7, 13). Weisenberg et al. (19) and Fox et al. (2) found no correlation between BMI and difficult intubation, while Lundstrøm et al., in a study including 91,332 patients, concluded that the correlation is weak (7, 13, 18). These discrepancies with our data can be explained by methodological differences and varying study designs. In fact, Ezri et al. studied morbidly obese predominantly female patients (BMI > 35 kg/m^2^) undergoing laparoscopic weight reduction surgery (7); however, the sample size of this study was significantly smaller (14723 subjects) compared to our study. The intubations were performed with size 3 Macintosh blade by only four experienced anesthesiologists (over 15 years of experience). In contrast, our study group consisted of adult patients of both sexes undergoing various surgical operations. The demographic data were widely variable (Table 2), and the intubations had been performed by personnel with a wide range of procedural skills (CA1 residents, trained CRNAs, and attending anesthesiologists). There was no standardization of the laryngoscope blade, since this was a retrospective study. Fox et al. studied 192 patients (146 women and 46 men) undergoing laparoscopic bariatric surgery (2). The BMI ranged between 35.8 and 82 kg/m^2^. All of the patients had been intubated by the same senior anesthetists after being placed in the beach chair position.

As in the case of previous authors, there were significantly more female patients included in the study group compared to males. Similarly, the study group, type of surgery, and the demographic data were confined to a specific type of patient undergoing a specific (weight reduction) surgery. In contrast, our patient group was representative for the general population treated in a university hospital. Lundstrøm et al. performed a study on an even a larger number of patients designed analogous to ours (18). Although the authors concluded that there was a significant but weak correlation between the BMI and risk of difficult intubation, they did not conduct a separate gender-based analysis of the BMI vs. difficulty of intubation. Another difference was that their study had been conducted in 14 anesthesia departments with unknown variability in patient groups and intubation skills. We had analyzed in the adult general surgical patients the relationship between the BMI and risk of difficult tracheal intubation in both genders. Our results clearly demonstrate that BMI is a strong indicator of difficult tracheal intubation among male patients. Mallampati score proved to be another strong predictor for both genders as well. Extensive research on fat distribution has been conducted in the field of sleep apnea, and those results may prove to be beneficial to research involving surgical airway management.

Effectively, Whittle et al. found a significant difference in the distribution of body fat of men and women (20). Using magnetic resonance imaging, they discovered that men have significantly more fat distributed to their trunk and palatal region, which may explain why sleep apnea is more common in men. Despite the small group sizes in the study, their results may constitute a possible explanation of our findings of sex-related difference in the BMI predictive value. Since our work had been designed as a retrospective study, we had to exclude 38.2% of the patients from our data analysis due to incomplete anesthesia chart records or not fitting in the inclusion criteria. Another limitation was the possible bias related to variability in intubation skills among the clinical personnel. We completely agree with Lundstrøm et al., who states that the possibility exists that a more experienced physician may be allocated for intubating a patient with higher BMI (18). Additionally, a patient may be scheduled for a fiberoptic intubation because of obesity, leaving this particular patient ineligible for analysis (18).

## Conclusion

In conclusion, our results indicate that the Mallampati score is a strong predictor of difficult tracheal intubation in adult patients (both genders) undergoing general surgical procedures. Our data shows that BMI is a reliable indicator of potential difficult tracheal intubation only in male surgical patients. Identifying the patients based on BMI assessment and Mallampati score may prevent possible consequences of difficult tracheal intubation and help to adopt an alternative approach for these patients. Such an approach will contribute to improvement in patient care and increase patient safety. Future prospective studies should aim to include controlled preoperative assessments and defined concept of difficult tracheal intubation.

## Conflict of Interest Statement

The authors declare that the research was conducted in the absence of any commercial or financial relationships that could be construed as a potential conflict of interest.
